# The ATP-dependent RNA helicase, DDX42 interacts with paxillin and regulates apoptosis and polarization of Ba/F3 cells

**DOI:** 10.1080/19768354.2019.1567580

**Published:** 2019-01-21

**Authors:** Sung Oh Sohn, Kee Oh Chay

**Affiliations:** Department of Biochemistry, Medical School, Chonnam National University, Jeollanam-do, Republic of Korea

**Keywords:** DDX42, paxillin, apoptosis, cytoskeleton, IL-3

## Abstract

Paxillin is a focal adhesion adaptor protein, heavily phosphorylated at multiple tyrosine residues, as well as at serine 273 (S273), and is known to be critical for cytoskeleton rearrangement and cell migration. We previously found that paxillin plays a regulatory role in IL-3-dependent survival of Ba/F3 cells, a mouse pro-B cell line. In this study, by using overexpressed His6 tagged-paxillin as a bait, we found that DDX42, a DEAD-box RNA helicase, interacted with paxillin, inhibited apoptosis, and promoted polarization of Ba/F3 cells. His6 tagged-paxillin was stably overexpressed in Ba/F3 cells, pulled-down from cell lysates with Ni^+^-NTA beads, and analyzed by one-dimensional SDS-PAGE followed by LC–MS. We found that DDX42 co-precipitated with paxillin, as demonstrated by western blotting analysis of His6 tagged-paxillin precipitates with anti-DDX42 antibodies and His6 tagged-DDX42 precipitates with anti-paxillin antibodies. In addition, we observed a preferential interaction of DDX42 with the paxillin mutant, S273A, compared to the S273D mutant. Furthermore, DDX42 overexpression in Ba/F3 cells delayed the apoptosis induced by IL-3 deprivation and promoted restoration of the elongated shape in Ba/F3 cells induced by IL-3 re-supply after a 6 h-deprivation. These results suggested that DDX42 interacts with paxillin and participates in IL-3-dependent cell survival, as well as in the cytoskeletal rearrangements underlying polarization of Ba/F3 cells.

## Introduction

The interaction of tissue cells with the extracellular matrix is critical for several cellular processes such as differentiation, migration, survival, and growth as well as for clinical conditions such as cancer metastasis (Shan & Zhang 2017; Ojalill et al. 2018). Cells express various combinations of integrin αβ heterodimers, functioning as extracellular matrix receptors. When cells are attached to the extracellular matrix, a protein complex, the focal adhesion, composed of a high number of signaling and structural proteins, is formed around the cytoplasmic tails of the clustered integrins. Focal adhesion not only plays a structural role connecting the actin stress fibers to the integrin receptors but also transduces bidirectional signals, inside-out and outside-in, contributing to the abovementioned physiological processes (Shen et al. 2012). Assembly and disassembly of focal adhesions are tightly regulated in migrating cells and occur at the front and rear side of migration, respectively (Duperret & Ridky 2013; Quizi et al. 2013).

Paxillin is a 68-kDa scaffold signal protein of focal adhesion, found to be a substrate of Src kinase in Src-transformed cells (Glenney & Zokas 1989). The structure of paxillin comprises two moieties: the N-terminal moiety contains five LD motifs, whereas the C-terminal moiety contains four LIM domains. LD motifs (LD*X*LL*XX*L) and LIM domains (zinc finger motifs) are regions involved in protein–protein interactions, conserved in all known paxillin homologs, such as Hic-5 (Deakin et al. 2012) and leupaxin (Vanarotti et al. 2016). LD motifs are essential for binding a variety of proteins such as FAK (Hildebrand et al. 1995), vinculin (Zhou et al. 2017), paxillin kinase linker (an ARF-GAP or PKL) (West et al. 2001), actopaxin (Nikolopoulos & Turner 2000), integrin-linked kinase (Moik et al. 2013), and bovine papilloma virus E6 protein (Sarode & Sarode 2014). The interaction with actopaxin, in particular, seems to be critical for paxillin connection to the actin stress fibers and for the regulation of actin rearrangements. The LIM2 and LIM3 domains are important for paxillin localization to focal adhesions by a phosphorylation dependent mechanism (Smith et al. 2013). LIM domains also participate in the interaction with PTP-PEST (Jamieson et al. 2005), regulating the balance between assembly and disassembly of focal adhesions. Paxillin also binds directly to integrins α_4_ and α_9_ and plays an important role in the regulation of cell migration (Deakin et al. 2009). Paxillin is phosphorylated at multiple tyrosine residues (e.g. Y31, Y40, Y118, and Y181), by FAK, CAK, ERKs, and/or Src (Hiregowdara et al. 1997; Ross et al. 2012; Hu et al. 2014). These phosphotyrosine motifs provide the binding sites for the SH2 domain of Crk I and CrkII (Downey et al. 2008). In particular, phosphorylation of the Y31 and Y118 residues is known to participate in actin cytoskeleton rearrangement during cell spreading (Romanova et al. 2004). Paxillin is also phosphorylated at serine/threonine residues by p21-activated kinase (PAK), JNK, and p38MAPK (Huang et al. 2004; Wei et al. 2013). S273, located in the LD4 motif, is phosphorylated by PAK, participates in the interaction with protein-coupled receptor kinase-interacting protein 1 (GIT1), activates the signaling complex GIT1-PIX-PAK, and regulates the dynamics of lamellipodia, as well as focal adhesions turnover (Nayal et al. 2006). Accumulating evidence suggests that paxillin plays a pivotal role in the integrin-mediated signal pathways underlying adhesion, migration, and anchorage-dependent survival of cells.

Ba/F3 cells, a pro-B cell line derived from mouse bone marrow, depend on IL-3 not only for survival and proliferation, but also to acquire their polarized shape. Notably, in the media supplemented with IL-3, Ba/F3 cells assume a characteristic elongated shape, which requires a polarized arrangement of the cytoskeleton (Romanova et al. 1999), whereas without IL-3 the cells lose the polarized shape, become rounded, stop at G1 point, and undergo apoptotic death *via* the activation of caspases (Chay et al. 2002). Paxillin is abundantly expressed in Ba/F3 cells and heavily phosphorylated by IL-3 (Romanova et al. 1999).

The RNA helicase enzyme superfamily comprises several DEAD box domain-containing members with a function in modulating RNA structure in an energy-dependent manner, thereby participating in various biological processes such as ribosomal assembly, trafficking, spermatogenesis, embryonal development, cell differentiation and growth, and cancer invasion (Suk et al. 2000; Abdelhaleem et al. 2003; Fuller-Pace 2013). DDX42 is a recently identified member of the DEAD box RNA helicase superfamily, the function of which has not been yet clarified. Uhlmann-Schiffler et al. (2009) reported that a C-terminal portion of DDX42 interacts with the pro-apoptotic factor, apoptosis-stimulating protein of p53 protein 2 (ASPP2), thereby inhibiting its action. Lin et al. (2008) reported that DDX42 interacts with NS4A, in Japanese encephalitis virus (JEV), and suppresses the initial immune responses during JEV infection.

In this study, we found DDX42 to interact with paxillin at a phosphorylation site including the S273 residue of paxillin. We also observed that DDX42 overexpression protected Ba/F3 cells from apoptosis induced by IL-3 withdrawal and regulated cell polarization by affecting IL-3-induced cytoskeleton rearrangements.

## Materials and methods

### Materials and reagents

Alexa Fluor 647-conjugated annexin V was from Molecular Probes (Eugene, OR, USA). N-acetyl-leucyl-leucyl-norleucinal (ALLN), N-acetyl-Leu-Leu-methioninal (ALLM), aprotinin, leupeptin, and 4-(2-aminoethyl)benzenesulfonyl fluoride (ABESF) were from Calbiochem (La Jolla, CA, USA). Antibodies against paxillin (610052), GIT1 (sc-13961), actin (A2066), and DDX42 (A303-354A) were from BD Korea (Seoul Korea), Santa Cruz Biotechnology (Santa Cruz, CA, USA), Sigma Aldrich Co. (St. Louis, MO, USA), and Bethyl Laboratories (Montgomery, TX, USA), respectively. The secondary HRP-labeled anti-mouse IgG and anti-rabbit IgG were from Amersham Biosciences. (Piscataway, NJ, USA). SuperSignal West Dura Extended Duration Substrate kit and BCA protein assay kit were from Thermo Scientific. (Rockford, IL, USA), Ni^+^-NTA agarose and FuGENE HD were from Qiagen (Hilden, Germany) and Promega (OH, USA), respectively. The 293T-based retroviral packaging cell line, 293 Plat-E, was kindly provided by Dr. Kitamura (Morita et al. 2000) from the University of Tokyo, Japan. An IL-3-producing (WEHI-3) and an IL-3-dependent (Ba/F3) cell lines were provided by the Bank for Cytokine Research (Chunbuk University, Korea) and Dr. Mushinski (NCI, ant the NIH, USA), respectively.

#### Cell culture and IL-3 deprivation of Ba/F3

The IL-3 producing WEHI-3 cells were maintained in RPMI 1640 medium supplemented with 10% heat-inactivated fetal bovine serum (FBS) and passaged 1:10 every 2–3 days. To obtain the WEHI-3-conditioned medium, WEHI-3 cells (10^7^) were seeded into culture medium (10^7^/100 mL) in a T175 culture flask and cultured for 4 days until the color of medium turned yellow. The IL-3 containing medium was harvested, centrifuged, filtered with a microfilter system (0.2 μm), and kept frozen at – 80°C. The IL-3-dependent mouse pro-B cell line, Ba/F3, was cultured in RPMI 1640 medium supplemented with 10% FBS and 10% WEHI-3-conditioned medium as a source of IL-3. Cells were passaged 1:10 every 2 days and maintained at a cell density of 10^5^–10^6^/mL. IL-3 deprivation was conducted as previously described (Chay et al. 2002). Briefly, cells were washed four times by centrifugation and resuspension in PBS at room temperature (pH 7.4). Next, 2 × 10^6^ cells were plated into 100-mm dishes containing 10 mL of pre-warmed IL-3-free medium and incubated in a CO_2_ incubator for 0–48 h. Plat-E cells were maintained in DMEM supplemented with 10% FBS, 1 μg/mL puromycin, and 10 μg/mL blasticidin.

### Vector construction and site-directed mutagenesis

A fragment containing the internalized ribosome entry site (IRES) signal sequence and enhanced green fluorescent protein (EGFP) cDNA was excised from the bicistronic expression vector pIRES2-EGFP (CloneTech; Palo Alto, California, USA) with the restriction endonucleases BglII and NotI, then cloned into pMX using the BamHI and NotI restriction sites, obtaining pMX-IRES2-EGFP, a retroviral bicistronic expression vector. A cDNA encoding the entire open reading frame of mouse paxillin-α was obtained by PCR amplification from a previously described construct (Chay et al. 2002) and cloned into pMX-IRES2-EGFP using the vector XhoI and EcoRI restriction sites. A QuikChange site-directed mutagenesis kit (Stratagene, La Jolla, CA, USA) was used for *in vitro* mutagenesis of S273, according to the manufacturer's instructions. A mouse DDX42 cDNA clone (IMAGE 5698965) was purchased from the I.M.A.G.E. consortium (http://imageconsortium.org/). The cDNA of the entire open reading frame of DDX42 was obtained by PCR amplification using specific primers (forward; 5′-TCTCTCGAGGCTGAAGTGGAGGATCAGGCTGC-3ʹ and reverse; 5′-CGCGGATCCCTAACTATCCCATCGGCTTTTC-3ʹ) and cloned into the XhoI and BamH1 restriction sites of *pMX-IRES2-EGFP* for the expression of His6-tagged DDX42. The D407 residue within the DEAD box of DDX42 was replaced with alanine to yield the D407A mutant. All clones were verified by DNA sequencing.

### Retrovirus packaging, infection, and cloning of cells stably overexpressing paxillin or DDX42

The day before transfection, 293 Plat-E cells (5 × 10^4^) were seeded into a 6-well plate in 2 mL of DMEM supplemented with 10% FBS. The next morning, the medium was replaced with 1 mL of pre-warmed fresh medium. Mock and cloned vectors were transfected into the packaging cells using the FuGENE 6 reagent (Roche Molecular Biochemicals, Germany), according to the manufacturer's instructions, and incubated in a CO_2_ incubator for 36 h to allow for retrovirus packaging. Ba/F3 cells (10^4^), in 1 mL of RPMI 1640 supplemented with 10% FBS, 20% WEHI-3-conditioned medium, and 8 μg/mL of polybrene, were added to the wells of the 6-well plate containing the transfected Plat-E cells, and co-cultured for 24 h to allow for virus infection. The infected Ba/F3 cells were moved into 100-mm tissue culture dishes and 9 mL of fresh RPMI 1640 supplemented with 10% FBS and 10% WEHI-3-conditioned medium were added. After culturing for an additional 24 h, cells were cloned into round-bottomed 96-well plates by limiting dilution. Clones exhibiting green fluorescence under the microscope were selected for expansion, and the expression of paxillin and DDX42 were determined by western blot analysis.

### Pull-down of His6-tagged paxillin and analysis by SDS-PAGE and LC-MS

Cells in 150-mm tissue culture dishes were washed twice with cold 20 mM imidazole-HCl (pH 7.4) containing 150 mM NaCl and resuspended in the same buffer. Appropriate amounts of ice-cold lysis buffer containing 20 mM imidazole-HCl (pH 7.4), 130 mM NaCl, 0.05% Tween-20, 1 mM sodium orthovanadate, 20 mM sodium fluoride. After brief sonication on ice and centrifugation for 15 min at maximum speed in a microcentrifuge, supernatants were assayed for protein concentration. To pull-down the His6-tagged protein, Ni^+^-NTA beads (15-μL bed volume, Qiagen) were added to 1 mL of cell extract (1 mg of protein), continuously inverted for 30 min at 4°C, washed four times with the lysis buffer, once with 2 M urea, and two additional times with the lysis buffer to remove residual urea. Proteins were eluted from the beads by resuspension in 150 mM imidazole, mixing with SDS-PAGE sample buffer, and heating at 100°C for 5 min, and then were separated with 8% SDS-PAGE gel and stained with a silver staining kit (Biosesang, Korea), according to the manufacturer's instructions. Protein bands exclusive of cell overexpressing the tagged protein, were excised from the gel and sent to the facility service in Yonsei Proteomic Research Center (Seoul, Korea) for LC–MS analysis. Briefly, the specimens were extracted by in-gel trypsinization and analyzed with an LTQ-XL mass spectrometer (Thermo Scientific, San Jose, CA). The data were analyzed with MASCOT (version 2.3.01) and the candidate interactors were searched in the UniProt mouse database.

### Western blotting

Cells in 100-mm tissue culture dishes were washed twice in PBS at 4°C and resuspended in cold PBS (pH 7.4). Appropriate amounts of ice-cold lysis buffer containing 0.5% Triton X-100, 0.5% Nonidet P-40, 0.5 mM EDTA, 0.5 mM EGTA, 150 mM NaCl, 10 mM Tris–HCl (pH 7.2), 10 μg/mL aprotinin, 10 μg/mL leupeptin, 1 mM ABESF, and 25 μM each of calpain inhibitors I and II (ALLN and ALLM, respectively) were added to the cell pellets. After brief sonication on ice and centrifugation for 15 min, supernatants were assayed for protein concentration, mixed with SDS-PAGE sample buffer, heated at 100°C for 5 min, and loaded onto an 8% Tris-glycine polyacrylamide gel. Western blots were performed using specific antibodies and signals were detected by chemiluminescence. A bicinchoninic acid (BCA) protein assay kit was used for protein assays, with bovine serum albumin as the protein standard (Smith et al. 1985).

### Annexin V conjugation assay

Cells (10^6^) were washed once with PBS containing 1 g/L sucrose and resuspended in 100 μL of binding buffer containing 10 mM HEPES (pH 7.4), 140 mM NaCl, and 2.5 mM CaCl_2_ (Andree et al. 1990). Cells were added with 5 μL of Alexa 647 (excitation: 647 nm, emission: 665 nm)-conjugated annexin V stock solution, incubated at room temperature for 15 min, and finally analyzed with the FACSCalibur system (BD PharMingen immunocytometry system; San Jose, CA, USA).

## Results

### Overexpression and pull-down of His6-tagged paxillin followed by proteomic analysis

**Figure 1.**
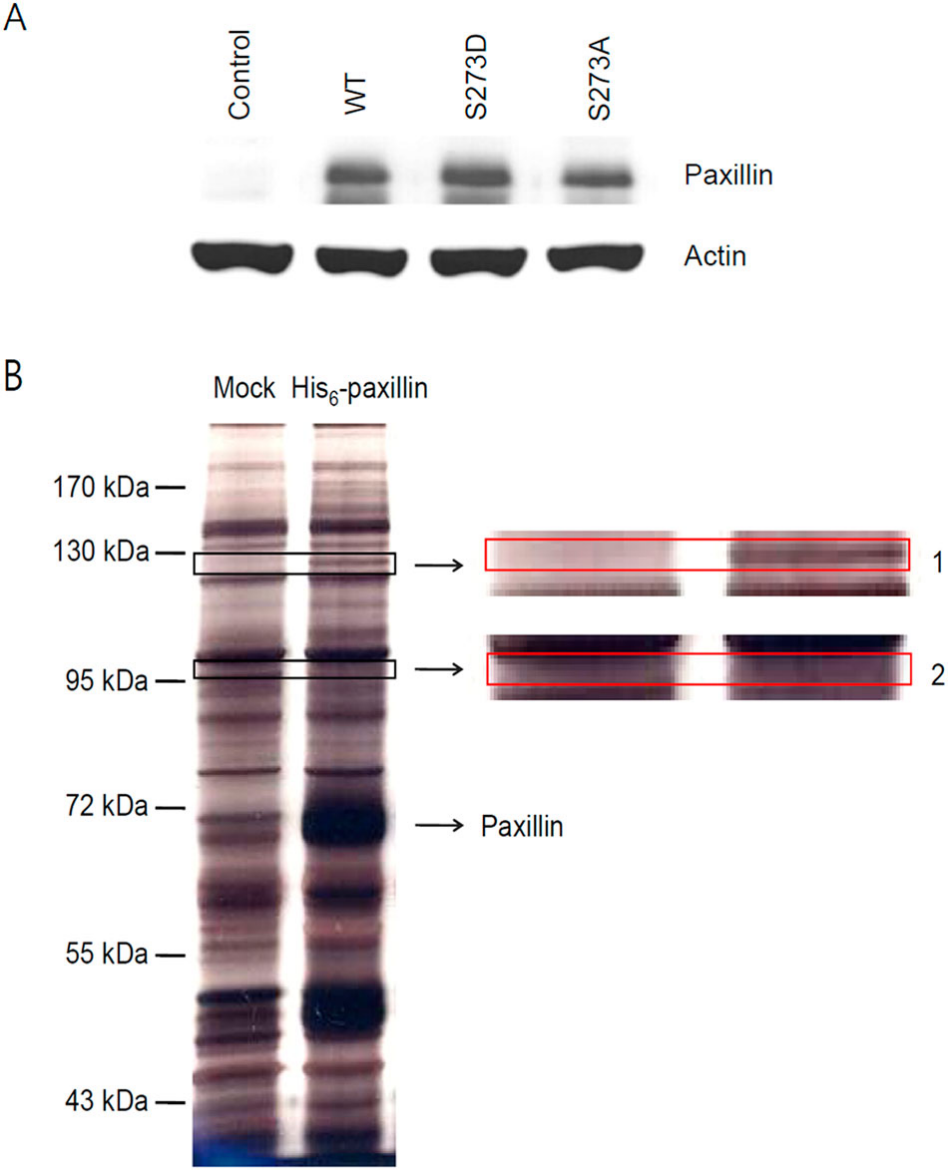
(A) Overexpression of His6-tagged wild-type and mutant paxillins. The expression of paxillin in non-infected Ba/F3 (Control) and in Ba/F3-derived cell lines overexpressing His6-tagged wild-type (Paxillin WT) and mutant paxillin (S273D and S273A) were compared by western blot analysis of cell extracts (20 μg protein) using anti-paxillin and anti-actin antibodies. (B) Comparison of protein bands of the pulled-down samples after electrophoresis and silver staining. Cell lysates from mock-transfected (Mock) and His6-tagged wild-type paxillin-overexpressing (His6-paxillin) cells, incubated with Ni^+^-NTA agarose beads, were loaded on an 8% polyacrylamide gel, and silver-stained. Differences in band patterns were observed in two regions (1 and 2), highlighted by rectangles, and are shown as magnified images on the right.

1. To verify His6-tagged paxillin overexpression, a clone with a fluorescence intensity comparable to that of the EGFP-overexpressing clone (mock-transfected with pMX-IRES2-EGFP) was selected from Ba/F3-derived cell lines overexpressing wild-type or mutant paxillin plus EGFP (Figure 1(A), Paxillin WT, S273A, S273D). The expression level of paxillin was compared by western blot analysis to that of endogenous paxillin in non-infected Ba/F3 (Figure 1(A), Control). The clones derived from paxillin-transfected Ba/F3 cells exhibited a much higher level of paxillin expression. The overexpressed wild-type paxillin was pulled-down with Ni^+^-NTA beads, separated by 8% SDS-PAGE, and stained, using a silver staining kit (Figure 1(B)). A few bands, in two distinct regions of the gel, were only observed in the lanes loaded with the paxillin-overexpressing cell lysates (Figure 1(B), His6-paxillin) and not in those of the mock-transfectants (Figure 1(B), Mock).

Gel slices were excised from the two aforementioned regions, dried, and sent for LC–MS analysis, by which a few potential paxillin interactors were identified (Table 1). GIT1 and GIT2, as well as αPIX, were already known to interact with paxillin and to be involved in actin cytoskeleton-dependent processes such as cell migration, spreading, formation of membrane ruffles, and cell division. The available data indicated that DDX 42 was the most probable candidate as a paxillin-interacting protein.

**Table 1.** Results of mass spectrometry analysis. Gel slices were cut out of two regions of each lanes in Figure 1(B) and sent for LC–MS analysis for peptide identification to the facility service in Yonsei Proteomic Research Center (Seoul, Korea).

Sample no.	Accession no.	Mass (Mr)	Control		Paxillin-WT		Description (taxonomy)
			Peptide score	Protein matches	Peptide score	Protein matches	
Region 1	Q810A7	102249	64.51	9	42.74	51	ATP-dependent RNA helicase DDX42
	Q6NV83	118716			52.77	3	U2-associated protein SR140
Region 2	Q5F258	84935	42.07	1	55.22	7	ARF GTPase-activating protein GIT1
	E9PVA6	85198			70.07	22	ARF GTPase-activating protein GIT2
	Q9JLQ2	79507			60.14	19	ARF GTPase-activating protein GIT2
	Q80Y52	85186			46.11	5	Heat shock protein 90, alpha
	Q80TG4	79110			40.35	3	MKIAA1261 protein (Transducin-like enhancer protein 4)
	A2AFI0	90345			42.53	3	Alpha-Pix

Note: This table is a brief summary of the results.

### Interaction of DDX42 with paxillin

**Figure 2.**
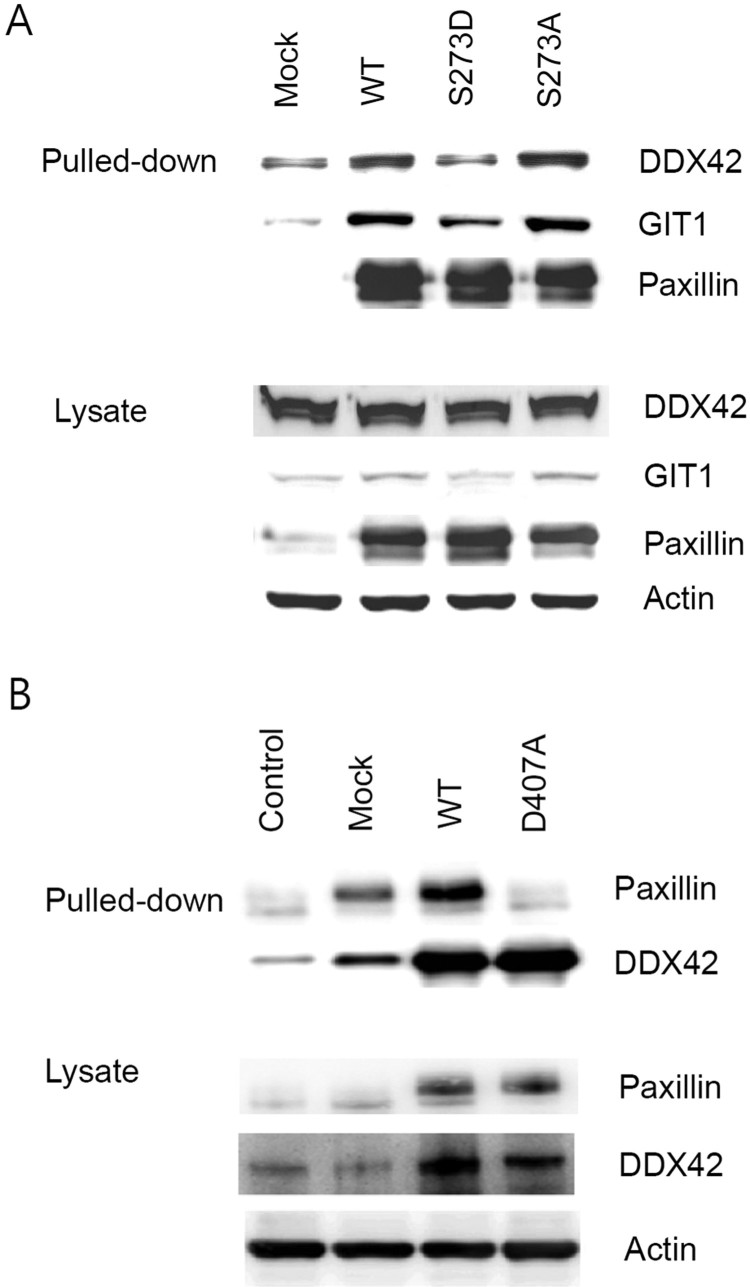
Interaction of DDX42 with paxillin. (A) His6-tagged wild-type (WT) and mutant (S273D and S273A) paxillin were overexpressed in Ba/F3 cells. The overexpressed proteins were pulled-down from each cell lysate with Ni^+^-NTA beads, and loaded on an 8% SDS-PAGE gel (Pulled-down). Pulled-down and co-precipitated proteins were detected by immunoblot with anti-DDX42, anti-GIT1, and anti-paxillin antibodies. DDX42 co-precipitated only with wild-type and S273A mutant paxillin, not with S273D. Whole cell lysates (20 μg protein) were also loaded on another gel and the indicated proteins were detected by immunoblot using the respective antibodies (Lysate). (B) His6-tagged wild-type (WT) and mutant (D407A) DDX42 were overexpressed and analyzed as described above (Pulled-down). Paxillin co-precipitated only with wild-type (WT), but not the DDX42 mutant, D407A. The amount of proteins was normalized by immunoblot of the lysates (Lysate).

The interaction between DDX42 and paxillin was verified by co-precipitation experiments, consisting of precipitation with Ni^+^-NTA agarose beads followed by western blotting in cells overexpressing His6-tagged paxillin or DDX42 (Figure 2). DDX42 was efficiently co-precipitated from cells overexpressing His6-tagged wild-type paxillin (Figure 2(A), WT) but not from mock-transfected cells (Figure 2(A), Mock).

Interestingly, DDX42 was also co-precipitated from cells overexpressing the His6-tagged paxillin mutant, S273A (Figure 2(A)) and, to a much lesser extent, from those overexpressing the His6-tagged paxillin mutant, S273D (Figure 2(A)). Western blot for GIT1, a protein known to interact with paxillin, showed a co-precipitation pattern similar to DDX42 (Figure 2(A), GIT1).

Consistently, paxillin was found to co-precipitate with DDX42 in cells overexpressing His6-tagged DDX42 (Figure 2(B), WT), but not in parental Ba/F3 (Figure 2(B), Control) or mock-transfected cells (Figure 2(B), Mock). Notably, paxillin was not co-precipitated in cells overexpressing the His6-tagged DDX42 mutant, D407A (Figure 2(B)).

### Effects of DDX42 overexpression on apoptosis and polarization of Ba/F3 cells.

**Figure 3.**
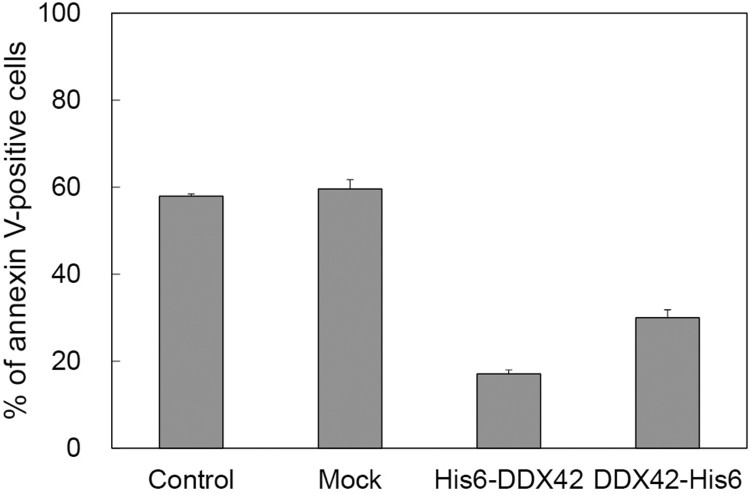
Protective effect of DDX42 overexpression against IL-3 withdrawal-induced apoptosis in Ba/F3 cells. Non-transfected (Control), mock-transfected (Mock), and His6-tagged DDX42 overexpressing cells (with both of N- and C-terminal His6 tag) were deprived of IL-3 for 24 h and analyzed by annexin V conjugation assay to determine the percentage of annexin V-positive cells. Each data point represents the average + S.D. of triplicate experiments.

Since paxillin overexpression in Ba/F3 cells inhibited apoptosis induced by IL-3 withdrawal, the possible role of DDX42 in this process was explored. Ba/F3 cells were deprived of IL-3 for 24 h, stained with Alexa 647-conjugated annexin V, and analyzed by FACS scan (Figure 3). Cells were divided into two groups: annexin-negative (live) and annexin-positive (dead) cells. More than half (58%) of the cells showed positive annexin signals (Figure 3, Control). Mock-transfected cells, only overexpressing EGFP, showed a degree of apoptosis similar to parental Ba/F3 cells (60%) (Figure 3, Mock). These results are perfectly consistent with those of previous reports (Chay et al. 2002). On the other hand, cells stably overexpressing EGFP plus N-terminal His6 tagged- or C-terminal His6 tagged-DDX42 (Figure 3, His6-DDX42 and DDX42-His6) exhibited a dramatic reduction in apoptosis (17% and 30%, respectively).

When grown under optimal conditions, most Ba/F3 cells (about 80–90%) display a characteristic elongated shape, reflecting a polarized intracellular distribution of actin ruffles and tubulin fibers (Romanova et al. 1999). Notably, this localized distribution of actin and tubulin was not observed in IL-3-deprived round-shaped Ba/F3 cells. We knew empirically that Ba/F3 cell polarization was very sensitive to various kinds of environmental changes, including physical and chemical factors, such as the availability of IL-3, pH, temperature, movement or vibration, perhaps even high cell density, all of which induce cell rounding. Even mild manipulations, such as pipetting during cell passages, may easily result in cell rounding. Notably, cells recover their elongated shape when kept for 1 or 2 h in the culture incubator.

**Figure 4.**
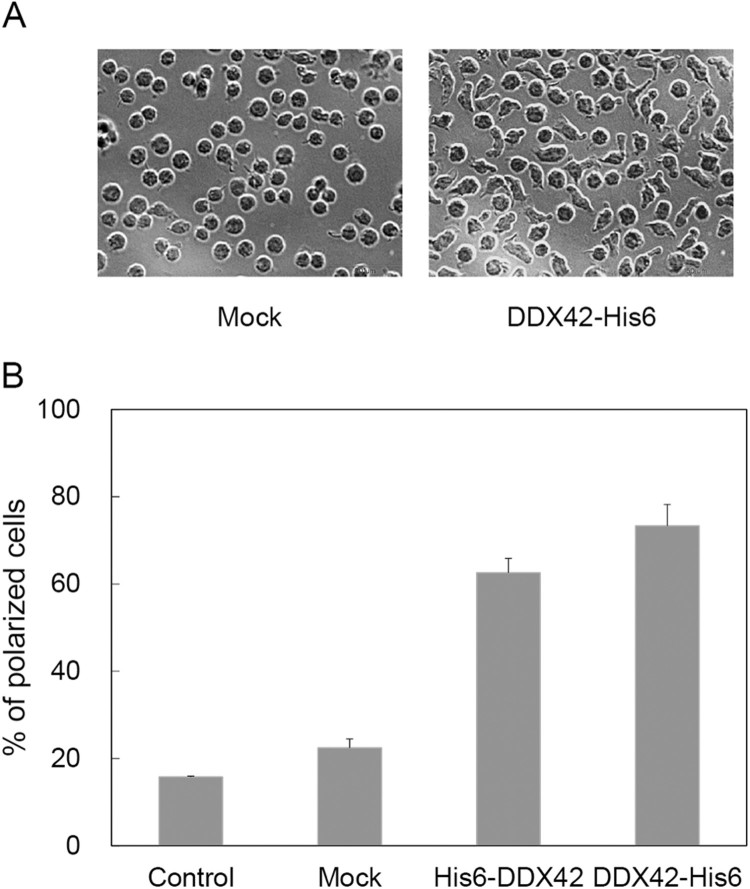
Effect of DDX42 overexpression on Ba/F3 cell polarization induced by IL-3. Untransfected (Control), mock-transfected (Mock), and His6-tagged DDX42 (His6-DDX42 and DDX42-His6) overexpressing cells were deprived of IL-3 for 6 h, re-supplied with IL-3, and incubated for 30 min in the CO_2_ incubator. (A) Photographs were taken with an inverted phase contrast microscope. (B) Cells with elongated shape were evaluated under a microscope and expressed as percentage of total 300 cells. Each data point represents the average + S.D. of triplicate experiments.

Occasionally, we noticed that Ba/F3 cells overexpressing DDX42 recovered a polarized morphology faster than non-transfected parental or mock-transfected Ba/F3 cells after routine manipulation. To explore this phenomenon, cells (10^5^) were washed four times with IL-3-free medium and cultured in 1 mL of the same medium for 6 h, after which they were supplied with 0.1 mL of pre-warmed IL-3-conditioned medium, and finally observed with an inverted microscope. We found that the simple operation of moving the culture dishes from the incubator to the microscope affected cell shape. Therefore, we fixed the cells within the incubator by very cautious addition of pre-warmed and pH-adjusted formaldehyde (3.7%, v/v) 30 min after the addition of IL-3. Photograms were taken with the microscope and the number of elongated and round cells were counted. Figure 4(A) showed the morphologies of mock-transfected (Mock) and DDX42-overexpressing (DDX42) cells 30 min after addition of IL-3-conditioned media. The proportions of elongated cells in parental Ba/F3 (Control), mock-transfected (Mock), N-terminal (His6-DDX42), and C-terminal (DDX42-His6) His6-tagged DDX42- overexpressing are shown in Figure 4(B). The overexpression of DDX42 strongly promoted the IL-3-induced polarization of Ba/F3 cells.

## Discussion

With the completion of the human genome project, the era of gene and protein discovery came to an end. However, the studies addressing protein–protein interactions and their biological roles have maintained their importance, because the functional meaning of these interactions cannot be unveiled by genomic approaches. The aim of this study was to identify paxillin interactors of which interaction are regulated in context of mammalian cell signaling, for example, by phosphorylation. Paxillin is known to be involved in several interactions, many of which are regulated by phosphorylation and are crucial for different cellular processes. (see Introduction for details). His6 tagged-paxillin was overexpressed in Ba/F3 cells (Figure 1(A)), pulled-down from the cell lysate with Ni^+^-NTA beads, washed with 2 M urea, and eluted with 150 mM imidazole, and the co-precipitated proteins were analyzed by one-dimensional SDS-PAGE (Figure 1(B)) followed by LC–MS (Table 1). The amount of eluted paxillin increased with the concentration of the imidazole solution up to 150 mM, as assessed by western blot analysis of the eluates with anti-paxillin antibodies (data not shown). Bead washing with 2 M urea allowed for substantial elimination of non-specific interactions (data not shown). We reasoned that specific interactions would be sufficiently stable to resist to relatively harsh conditions such as 2 M urea. We identified DDX42 as the most probable candidate paxillin interactor, and used western blotting to evaluate the expression of DDX42 in 7 commonly utilized cell lines (Ba/F3, NIH3T3, COS-7, ICE-18, RIE, 293T, HeLa; data not shown). All tested cells exhibited a similar level of expression, possibly reflecting a role of DDX42 as a housekeeping protein essential for survival or proliferation.

Next, we used His6-tagged paxillin- or DDX42-overexpressing cells and precipitation/western blotting experiments using anti-DDX42 or anti-paxillin antibodies, respectively, to demonstrate the interaction between the two proteins. Notably, paxillin did not co-precipitate with the DDX42 mutant (D407A), in which the DEAD box was affected by the mutation. DDX42 co-precipitated with the paxillin mutant, S273A, but not with the other paxillin mutant, S273D. Paxillin interactions involving S273 were found implicated in many cellular processes such as regulation of migration speed and formation of membrane ruffles. We concluded that the DEAD box of DDX42 and the S273 residue of paxillin are essential elements, and that S273 phosphorylation might negatively affect the interaction between the two proteins.

We explored the cellular function of DDX42. We found that the overexpression of DDX42 protected Ba/F3 cells from apoptosis induced by IL-3 withdrawal. In our previous study, we demonstrated that overexpression of paxillin inhibited the apoptosis of Ba/F3 cell induced by IL-3-withdrawal (Chay et al. 2002) and that re-addition of IL-3 induced paxillin tyrosine phosphorylation (Romanova et al. 2004). Recently, we found that tyrosine phosphorylation of paxillin at each Y31, Y40, Y118, and Y181 residue was required for paxillin inhibition of Ba/F3 cell apoptosis (in press). Overexpression of paxillin mutants (Y31F, Y40F, Y118F, Y181F) promoted rather than inhibited apoptosis of Ba/F3 cells in IL-3-deprived condition. So, we speculated that paxillin tyrosine phosphorylation is a critical event in IL-3/IL-3R-mediated survival signal pathway.

In this study, we also found that overexpression of DDX42 promoted the changes in morphology, likely accompanied by cytoskeletal rearrangements, to make polarized intracellular distribution of actin and tubulin, induced by re-addition of IL-3 to the cell depleted of IL-3 (Figure 4). Our previous (Chay et al. 2002) and current results collectively show that paxillin and DDX42 exerted the same effects on apoptosis, yet they exhibited different effects on cell polarization: while overexpression of WT or mutant (S273D) paxillin did not promoted cell polarization (data not shown), DDX42 overexpression did. Based on these, we speculated that the interaction between DDX42 and paxillin may not be the common upstream event of two bifurcated pathways for cell survival and polarization which have been initiated by single stimulation, re-addition of IL-3 in this system. So, we hypothesized that there might be a signal pathway regulating cell polarization through modulation of DDX42 independently of survival signal pathway in which both DDX42 and paxillin are involved. However, we could not exclude the possibility that DDX42 regulates cell polarization in a manner independent of IL-3/IL-3/R signal pathway.

**Figure 5.**
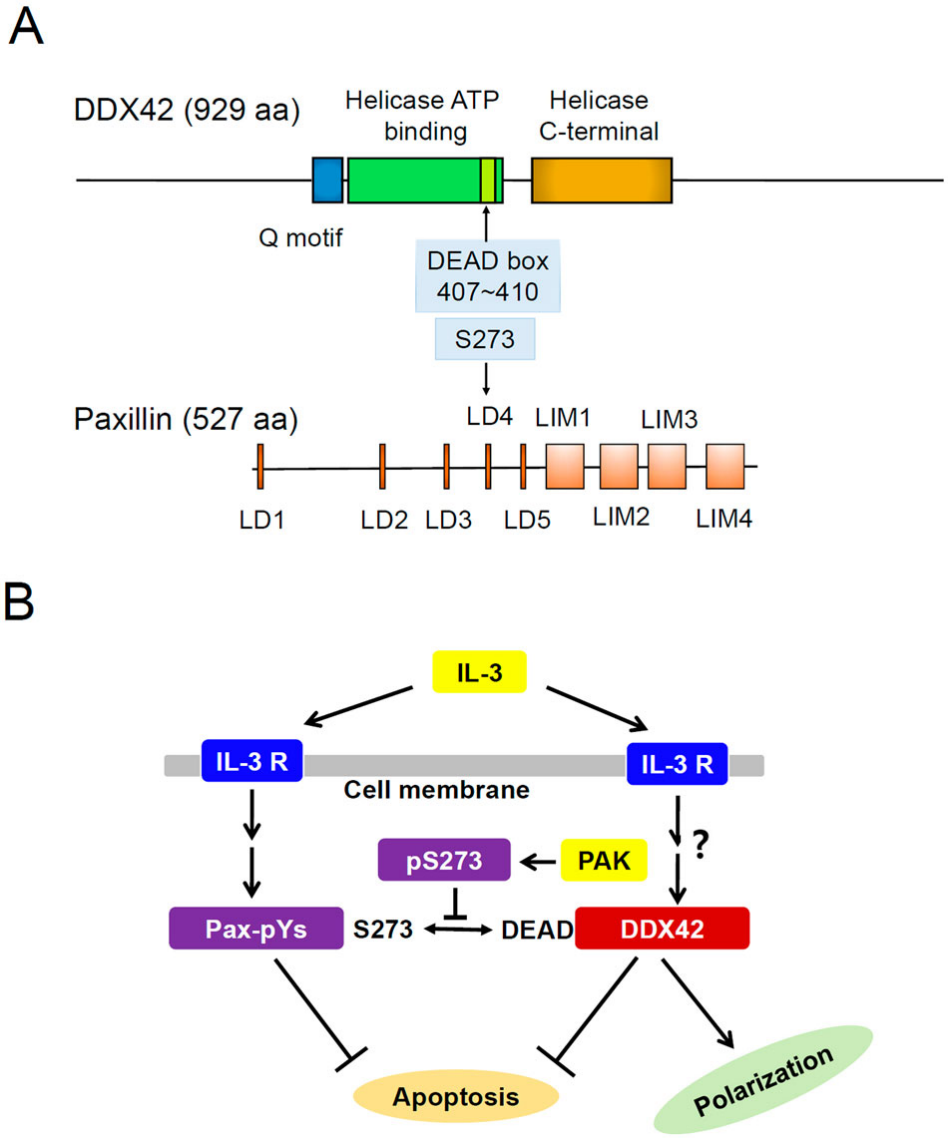
DDX42 and paxillin interact with each other through the DEAD box and S273, respectively. Both proteins showed anti-apoptotic effects when overexpressed in Ba/F3 cells. (A) Illustration of DDX42 and paxillin structure showing their main motifs and domains. (B) hypothetical model of the impact of the DDX42/paxillin interaction on cell apoptosis and polarization. Phosphorylation of S273 might abolish not only the anti-apoptotic action of paxillin but also its interaction with DDX42. Mutational inactivation of the DEAD box in DDX42 also prevented the interaction with paxillin.

Figure 5 shows the domain structures of DDX42 and paxillin and a possible model explaining how their interaction may be involved in apoptosis and polarization in Ba/F3 cells. DDX42 can be divided in three portions: A DAED box containing helicase domain in the middle and N- and C-terminal auxillary domains whose functions remain undetermined. A helicase domain, especially the DAED box, is highly conserved throughout all members of the RNA helicase superfamily (Sloan & Bohnsack 2018). Both auxillary domains have been reported to function through protein–protein interaction (Lin et al. 2008; Uhlmann-Schiffler et al. 2009; Jankowsky 2011; Putnam & Jankowsky 2013). RNA helicases usually require specific protein cofactor not only for activity but also for specificity (Heininger et al. 2016; Sloan & Bohnsack 2018). When bound to these cofactors, RNA helicases play roles in modulation of RNA structue as chaperones (Uhlmann-Schiffler et al. 2006; Jarmoskaite & Russell 2011, 2014). Mutational inactivation of the DEAD box in DDX42 prevented the interaction with paxillin, indicating that RNA-modulating function of DDX42 might be required for the interaction. Conclusively, these results may shed light on the function of DDX42 in IL-3 receptor-mediated signal transduction, critical for survival, movement, and proliferation of Ba/F3 cells.
